# Professionals' Views on Social Care Planning and Provision for People With Young‐Onset Dementia and Their Families in England: Findings From the DYNAMIC Study

**DOI:** 10.1002/gps.70155

**Published:** 2025-09-20

**Authors:** Catherine Quinn, Helen Young, Vasileios Stamou, Kate Gridley, Clare Mason, Jan Oyebode

**Affiliations:** ^1^ Centre for Applied Dementia Studies University of Bradford Bradford UK; ^2^ Older Adults' Social Care Research Group University of York York UK

## Abstract

**Objectives:**

Access to appropriate social care post‐diagnosis is crucial for people with young onset dementia (YOD) and their families. Yet care is hugely variable, frequently lacking, and poorly coordinated. We aimed to establish levels of awareness, knowledge, and practice among professionals regarding social care provision for people with YOD.

**Methods:**

A short survey (24 items) was developed based on previous research and through consultation with experts by experience and the project steering group. The aim was to establish awareness, knowledge, and practice among professionals regarding social care needs, care planning, and provision for people YOD. The survey was available online.

**Results:**

There were 139 responses from health and social care professionals. A wide range of situations triggered referrals to social care, illustrating the holistic impact of YOD. However, most referrals were triggered by crises rather than a proactive approach and were in response to carers' needs rather than those of the person with YOD. Referrals for advice and guidance around financial impacts were common. Most respondents reported there was no agreed care pathway and no YOD‐specific guidelines in their service. Areas of good practice in social care provision included: person‐centred and reablement based approaches; multi‐disciplinary and multi‐agency working; support from peers and the third sector; seamless care pathways and dedicated YOD services; support for carers; and personal budgets.

**Conclusions:**

Staff need accurate knowledge and awareness of specific YOD‐related issues to provide effective social care for those with YOD and their families. The current system tends to be reactive at point of crisis, whereas proactive planning and provision could pre‐empt crises and provide more effective support. YOD‐specific care pathways and guidance are needed to improve social care for this population.

## Key Points

- People with YOD and their families have specific social care needs, yet services for people with dementia are often designed with older people in mind.
- Over a third of health and social care professionals surveyed reported lacking the knowledge they needed about YOD and only around 10% expressed full confidence in supporting people with YOD and their families.
- Referrals to social care were typically due to concerns about the carer. Other reasons included financial needs, support with self‐care, and housing/accommodation needs.
- Areas of good practice in social care planning and provision involved person‐centred and reablement‐focused approaches, multi‐disciplinary working, dedicated YOD services and support for carers.

## 1 Introduction

Approximately 982,000 people are currently living with dementia in the United Kingdom (UK; 1). Young‐onset dementia (YOD) is defined as dementia with symptom onset under 65 years of age [1]. It is estimated that around 70,800 people, 7.5% of those diagnosed with dementia in the UK, are living with YOD [2, 3].

People with dementia of all ages experience challenges in accessing social care, related to individual aspects of the person with dementia and/or carer, such as willingness to seek and accept help, and systemic issues, such as the nature and organisation of services available [4, 5]. Social care needs, including for company, social opportunities, and meaningful activity, are among the most common unmet needs [6] and social care is often not adapted to meet the needs of those with rarer types of dementia some of which have a younger age of onset [5]. Studies have not directly compared social care needs of those with dementia by age. One study [7] found family carers of those with YOD did not differ from carers of those with late onset in terms of psychological well‐being, confidence or social support but contended with a different context as they were more likely to be balancing employment with caring. However, people who are younger experience distinct challenges compared with those diagnosed at a later age, arising from differences in individual and family life‐stage, lifestyle and overall health between those who are in middle‐age and those who are older. The stigma of being diagnosed at an early age with a condition associated with later life may lead to social isolation [8]. People may have young children to support and mortgage obligations. Those in employment may have to give up work [9] with loss of income creating financial difficulties [10], particularly as they are unlikely to be at an age to receive their pension [11]. Other family members may have to take on employment or work longer hours to provide some financial stability [12, 13], and have to juggle work with caring and other responsibilities such as childcare [11, 14]. Loss of employment may also leave the person without meaningful activity and lacking a sense of purpose [15]. Being younger means that people with a YOD diagnosis are more likely to be physically fit and need opportunities for physical activity. Taken together it becomes evident that those affected by YOD require age‐specific needs‐appropriate services, particularly with respect to social care and support.

Age‐appropriate social care services are important for the well‐being of both people with YOD and their family members [16, 17, 18]. Access to such services can be hindered by different factors. For instance, delays in receiving a YOD diagnosis are commonly reported [11, 16]. As YOD is rarer than late onset dementia, individuals affected by the condition may delay seeking help for symptoms due to a lack of awareness [19]. Professionals may misattribute dementia symptoms at a young age to other factors such as psychological disorders, and generally find it challenging to provide an accurate diagnosis due to the wide range of YOD subtypes [16, 19]. Once a YOD diagnosis is provided, people with YOD and their families may struggle to find age‐appropriate social care and services for which they are eligible [20]. Existing social care dementia services tend to be designed for older people, who face different challenges. In a survey of 233 people with YOD and their families, only just over a quarter had received home/social care [17]. Social care professionals in generic dementia services may lack the specialist knowledge and skills required to best support people with YOD through, for example, age appropriate help with finances [11, 21], suitable activities for fit middle‐aged people [20], opportunities for meaningful activity in place of paid employment [16], or age‐appropriate social contact and peer support [22]. Indeed, evidence suggests that people affected by YOD are more satisfied with their care when this is delivered by specialist YOD services [17, 23]. Social care providers themselves have recognised challenges in navigating and sourcing age‐appropriate YOD services and report that there are limited opportunities to be flexible and responsive to needs [24].

There is a need to better understand social care provision for people with YOD and their families, and how it can be improved. The aim of this study was to establish levels of awareness and knowledge among professionals regarding social care needs for people with YOD, and to better understand current social care‐related practice and pathways for this group.

### 1.1 Design

This research was undertaken as part of the DYNAMIC study [25], which aimed to establish current practice in English social care for people with YOD and co‐produce evidence‐based resources for improvement. The study included interviews with people living with YOD and their families, a survey of staff and the co‐production of resources to improve social care. This article focuses on the staff survey which involved the distribution of a cross‐sectional online survey to staff with a role in or awareness of adult social care planning, provision, management, or commissioning in England. The study was approved by the ethics committee of the University of Bradford and the Health Research Authority Social Care REC (reference 23/IEC08/0034).

### 1.2 Participants

Participants were staff working in England with a role in or awareness of adult social care planning, provision, management, or commissioning. Participants had to be working in England as the English social care system is different from other parts of the UK. Staff could be employed by Local Authorities, third sector, healthcare, private sector, or integrated care boards. They did not have to work directly with people with YOD, but needed to have a remit that *could include* people with YOD or their carers.

### 1.3 Data Collection

We developed a short survey to explore social care for people with YOD. Initial survey items were informed by previous research about the experiences of people with YOD in accessing support [11, 16] and from our previous experience of developing questions for a survey on YOD service delivery [17]. We also consulted with the experts‐by‐experience on our project team and members of our project steering group which included professionals working within social care. To ensure appropriate survey length, wording, and content, we sought feedback on a draft version of the survey from nine people in social care roles. This resulted in amendments to the survey questions. We then piloted a revised version using cognitive interviewing [26] with four individuals working in social care to explore their understanding of the survey questions. This resulted in further modifications before the survey was finalised. The final version consisted of 24 core questions (see Supplementary materials) and primarily comprised of closed‐ended questions; including questions about employing organisation, job title, number of people with dementia and people with YOD seen over a 3‐month period, and aspects of job role. Two Likert‐type questions on respondents' knowledge and confidence to work with people with YOD, respectively, were included. Open‐ended questions were used to enable participants to elaborate on their responses; for example, to expand on situations that triggered referrals for social care assessments. All questions were optional, so participants could omit any questions they did not wish to answer.

The survey was available online, with an option for participants to request a paper version (no‐one took up this option). It was publicised through social media, key contacts of the research team, and networks such as the Association of Directors of Adult Social Services, CHAIN (Contact, Help, Advice and Information Network), National Academy of Social Prescribing, Social Care Institute for Excellence, and Young Dementia Network (hosted by Dementia UK). The survey was available over a period of 28 weeks, from November 2023 to May 2024. We left the survey open for six months to enable us to gain as full a picture as possible of social care across England. We closed it when there was a noticeable decline in the number of responses and also to allow data analysis to be completed to inform the next stage of the project (outlined in the protocol [25]).

### 1.4 Data Analysis

Quantitative data were analysed descriptively using SPSS v.29. Responses to the open‐ended questions were imported into Excel and analysed using content analysis [27] to identify common categories across participants' responses. Initial content analysis was undertaken by a female professor, educated to PhD level and a qualified clinical psychologist, who has significant experience in qualitative analysis and undertaking YOD‐related research. The resultant analysis was then checked by a female associate professor, educated to PhD level and with significant experience in qualitative analysis and dementia care research. Emergent categories were discussed with the wider research team, including experts by experience, to ensure rigour, plausibility, and relevance [28].

## 2 Results

In total, 139 people completed the survey. Demographic characteristics are summarised in Table 1. The majority of respondents were female (83.5%) and most commonly working in local authorities (34.5%), mental health trusts (25.9%), or third sector organisations (18.7%). Respondents were involved in referrals for social care assessments, carer assessments, and social prescription (Table 2).

**TABLE 1:** Demographic information about respondents.

Characteristic	(*n*, %)
Sex	
Male	17 (12.1%)
Female	117 (83.6%)
Non‐binary	1 (0.7%)
Age	
20–30	10 (7.1)
31–40	14 (10)
41–50	44 (31.4)
51–60	50 (35.7)
61+	18 (12.8)
Ethnicity	
White British	96 (68.6)
Asian/Asian British: Indian	2 (1.4)
Asian/Asian British: Pakistani	6 (4.3)
Black/Black British: Caribbean	4 (2.9)
Black/Black British: African	2 (1.4)
Black British	1 (0.7)
Mixed	2 (1.4)
Mixed White & Asian	1 (0.7)
Region in England	
North East	12 (8.6)
North West	12 (8.6)
Yorkshire & the Humber	56 (40)
West Midlands	8 (5.7)
East Midlands	7 (5)
East of England	8 (5.7)
Greater London	9 (6.4)
South East	14 (10)
South West	13 (9.3)
Type of work location	
Urban	70 (50)
Semi‐rural	30 (21.4)
Rural	13 (9.3)
All	24 (17.1)
Job title	
Social worker	16 (11.5)
Support worker	9 (6.5)
RMN	6 (4.3)
Admiral Nurse	6 (4.3)
Dementia specialist nurse	3 (2.2)
Other nurse	8 (5.8)
Other health or care professional	33 (23.7)
Commissioner	10 (7.2)
Dementia advisor	7 (5)
No profession stated	31 (22.3)
Other	10 (7.2)
Length of time in current job role	
Under 1 year	20 (14.4)
1–2 years	27 (19.4)
3–5 years	31 (22.3)
More than 5 years	61 (43.9)
Type of service working within	
Local authority	48 (34.3)
Mental health trust	36 (25.7)
Primary care	10 (7.1)
Third sector organisation (charity)	28 (20)
Hospital trust	13 (9.3)
Integrated care board	7 (5)
Other	19 (13.6)

**TABLE 2:** Experiences of social care provision for people with YOD and their families.

Question	(*n*, %)
Approximately how many people with dementia have you had contact with over the previous 3 months?	
0	9 (6.5)
1–10	33 (23.7)
11–20	17 (12.2)
21–50	40 (28.8)
More than 50	35 (25.2)
Unsure	3 (2.2)
Approximately how many of these people had young‐onset dementia?	
0	18 (12.9)
1	24 (17.3)
2–5	49 (35.3)
6–10	17 (12.2)
More than 10	22 (15.8)
Unsure	6 (4.3)
Where you work, is there an agreed care pathway for people with YOD and their families?	
Yes	39 (28.1)
No	54 (38.8)
Unsure	42 (30.2)
Where you work, do you have a written policy that gives guidance on how to work with people with young onset dementia and their families?	
Yes	15 (10.8)
No	79 (56.8)
Unsure	43 (30.9)
Do you refer people with YOD/families for social care assessments?	
Yes	98 (70.5)
No	35 (25.2)
Do you refer people with YOD/families for carer assessments?	
Yes	99 (71.2)
No	34 (24.5)
Do you refer people with YOD/families for social prescription?	
Yes	72 (51.8)
No	59 (42.4)
Do you undertake or have oversight of social care assessments, carer assessments or social prescription for people with YOD/families	
Yes	60 (43.2)
No	77 (55.4)
Do you devise care plans or provide social care for people with young‐onset dementia/families	
Yes	72 (51.8)
No	65 (46.8)
Confidence in ability to undertake social work planning and provision for people with YOD and families	
Very confident	13 (9.4%)
Quite confident	56 (40.3%)
Not very confident	28 (20.1%)
Not at all confident	8 (5.8%)
Not part of my role	30 (21.6%)
Knowledge of social work planning and provision for people with YOD and families	
Very knowledgeable	10 (7.2%)
Quite knowledgeable	68 (48.9%)
Not very knowledgeable	48 (34.5%)
Not at all knowledgeable	8 (5.8%)
Don't know	1 (0.7%)

Only 28.1% respondents indicated there was an agreed care pathway in their organisation for people with YOD and their families (Table 2). Similarly, only 10.8% had a written policy at their place of work on working with people with YOD and their families. Over a third (34.5%) of respondents rated themselves as not very knowledgeable about undertaking social care planning and provision for people with YOD and their families, and only 9.4% expressed full confidence in this respect.

### 2.1 What Situations Triggered Referrals for a Social Care Assessment for People With YOD or Their Families?

Responses (Table 3) indicated that referral for social care assessment were often only made when there were crises or safeguarding issues:

*“Generally at a point of crisis where families are struggling within their carer roles or change in dementia that causes strain on all involved. Safeguarding concerns are also a trigger point.”*

**TABLE 3:** Situations that triggered referrals for social care assessments.

Category	n^a^
Pathway for the referral	
Crisis/safeguarding issues	54
Referrals from other services	13
Initiation by person with YOD or carer	8
Change in person's health/hospitalisation	5
Type of care need	
Concerns about a carer	59
Self‐care activities	24
Financial issues	21
Housing/accommodation	17
Socio/occupational needs	12
Behaviour‐related needs	8
Cognitive changes	5
Children and family issues	5
Mental health needs	2

^a^ Refers to number of extracts coded under the category.

Other routes to social care assessments were: routine referrals from other services, initiation by person with YOD or their carer, a change in the person's health or hospitalisation.

Respondents identified the type of care needs that triggered social care assessments. The most common category was concerns about the carer (Table 3). This could be about changes in the carer's circumstances, such as being ill or needing hospital treatment, or the carer being stressed and struggling to meet the person's needs, leading to risks of breakdown in care arrangements:

*“Where the family carer can no longer cope with them at home and want to arrange permanent care”.*

It was noted that carers who were in employment could struggle to combine working with caring. Changes in the ability of the person with YOD to undertake self‐care activities, such as washing and dressing, could also prompt social care assessments as the person might need more formal support with these types of tasks:

*“The person not being able to take medication, wash and dress or make themselves a small meal independently”.*

Decline in cognitive functioning or changes in mental health or behaviour‐related needs sometimes triggered referrals for social care, especially if behaviour changes were causing concern to others. Wider family issues also led to social care referrals when support was needed for children or other family issues.

Some referrals for social care were related to financial issues. Support might be needed because the person was struggling financially, possibly due to loss of earnings. They might need help with financial planning or putting in place measures such as lasting powers of attorney, or need assistance with accessing benefits or because their own funds have depleted and they require local authority support to pay for care:

*“When finances are stretched and funding private support is no longer an option”.*

The individual with YOD might have social/occupational needs due to social isolation or lack of social stimulation and be referred for help accessing support groups, day care, or group activities.

Housing issues also triggered social care assessments if the home the person was living in was not suitable. The person may need help with home adaptations, due to increased care needs and declining function for example mobility. Individuals living with YOD might need their place of residence assessed before being discharged from hospital, or they might need to move into full‐time care if they can no longer manage at home.

### 2.2 How Could Access to Social Care for People With YOD be Improved?

Respondents identified the need for better awareness of YOD and YOD services (*n* = 64, n refers to the number of extracts coded). This encompassed raised awareness of YOD and individual's needs in both professionals and wider society, which would help reduce stigma. There also needed to be better provision of information about YOD and increased awareness of services for people with YOD and their families:

*“Support needed on who, how, when and where support can be accessed”.*

It was recognised that there needs to be better commissioning and care pathways for people with YOD and their families (*n* = 46). This included both a higher number and better planning of YOD‐specific services, as well as improvements to existing care pathways:

*“Better planning to shape services and approaches. Real planning around younger people”.*

*“* *A clear pathway for someone with young onset as their needs might be more complex”.*

Many respondents stated that there should be better community support (*n* = 65). This included having more age‐appropriate services, such as age‐specific peer support and activity groups for people with YOD. It was recognised that often working‐age people with dementia were directed inappropriately to older adult services:

*“* *People tend to be mixed with older people, which can be scary for younger service users and can also isolate them if they have a limited amount in common with their peers”.*

It was also suggested that reduced waiting times for diagnosis and social care services would lead to better community support, as needs would be assessed and addressed in a timely and more efficient manner. There was also a call for better support around finances, such as help with accessing benefits:

*“There are no/limited services to support with financial advice and benefits claims for those under 65 in my area”.*

Respondents identified a need for better short‐stay, residential and home care services for people with YOD, including more specialised YOD home care, and better support to find YOD‐specific services:

*“The search for appropriate respite and permanent care needs improving, carers are given long lists of homes who often dismiss younger people with dementia when rung. Also what homes do take younger people seem to have little appropriate stimulus”.*

Support for carers and the wider family were cited as requiring improvement (*n* = 10):

*“A clearer understanding of the impact dementia has on carers through extensive training of social care staff and more practical help available”.*

These suggestions recognised that family carers are often in employment and may need flexibility in provision (e.g. availability of services in the evening). Support was also needed for the wider family, including children, who may have a role in providing care:

*“Child/adolescent support, often young children are default carers”.*

### 2.3 Areas of Good Practice

Respondents also provided examples of areas of good practice in social care planning and provision. These focussed on: person‐centred and reablement based approaches (*n* = 31); multi‐disciplinary and multi‐agency working (*n* = 24); support from peers and the third sector (*n* = 19); seamless care pathways and dedicated YOD services (*n* = 11); specific support for carers (*n* = 11); and personal budgets (*n* = 5). These often aligned with the areas that respondents had identified as needing improving.

Person‐centred and reablement based approaches focussed on having individualised care packages and support that were age‐appropriate for the individual. These focussed on the needs of the individual and their supporter, promoting enablement and supporting individuals with their daily life:

*“Taking time to properly understand in individual's needs, ensuring they have a voice in their care, getting perspectives of carers and family, being open and respectful, building trust”.*

Multidisciplinary or multiagency working was seen as an area of good practice as it fostered collaborative working relationships and facilitated the sharing of information. Respondents gave examples of healthcare professionals undertaking joint working with adult social care professionals or developing links with voluntary agencies. Multidisciplinary team meetings brought together people with different expertise and could also include someone with specialist knowledge of YOD:

“*In my area we have a young onset dementia social worker which I work closely with. This specific speciality is paramount for patients as there is generally a lack of understanding in the complex social care needs for people with young onset dementia*”.

Respondents gave examples of dedicated YOD services that worked well to meet individual's needs. They also described ways of removing barriers for people transitioning to different parts of the pathway, such as how people accessed services:

“*Our day service for people with younger onset dementia is accessed directly, without social worker assessment*”.

Discussions on personal budgets highlighted the value of direct payments and the use of personal budgets to pay for things or to employ a personal assistant.

Some respondents had set‐up their own support groups as there weren't any in the area. They recognised that to work these needed to be flexible so, for example, running in the evenings after work. The benefits of groups for carers were that they provided information and support, but also opportunities for respite. Similarly, peer support groups or buddy schemes for people with young onset dementia provided opportunities to socialise and meet others.

*“Young onset peer buddy scheme. Fantastic group of young people living with dementia that are prepared to challenge services and support others on their journey with dementia”.*

## 3 Discussion

This is one of the first studies to explore social care provision in England for people with YOD. Most respondents were professionals involved in referring people for social care assessments or carer assessments. Over half devised care plans or provided social care for people with YOD directly, yet they varied in their knowledge and confidence in working with people affected by YOD. At their place of work, only 28.1% of participants reported there was an agreed care pathway and only 10.8% had a policy on working with people with YOD and their families. The findings highlight the shortcomings of social care services and how access to social care could be improved.

Crisis and safeguarding issues constituted the primary reasons for referrals for social care assessment. This finding indicates that the system is reactive, rather than working towards preventing crises from occurring. Proactive social care input could delay and potentially prevent development of situations that carry a high risk of breakdown in care arrangements. The most common care needs triggering social care referral were concerns about the carer. Professionals reported that carers had particular issues trying to combine care with paid employment and other family responsibilities. Carers have previously reported challenges in balancing work roles and caring [29], identifying the need for flexibility in their workplaces and ability to take time off work when necessary [30]. Some carers have reported that using respite services has enabled them to keep working [31]. Collaborations between service providers and employers could facilitate such arrangements and enable carers to continue providing informal care while maintaining employment.

Another trigger for social care referral was financial issues. If a person with YOD or carer has to reduce working hours or stop working completely this can cause financial hardships [11, 20, 29] and this can also affect dependent children [32]. In later life, the person with dementia and spousal carer have usually already retired so these issues are less acute. People with YOD may need support with accessing financial benefits or completing complex funding assessments [8, 33, 34] that often differ from those which apply in later life. It is important that advice and support with financial planning and entitlements are offered by YOD‐specialists early after diagnosis, to reduce the financial impact of the condition.

Over a third of respondents rated themselves as not very knowledgeable about YOD and only 9.4% expressed full confidence in working with people with YOD and their families. Raising awareness amongst professionals and those affected by YOD about the nature of the condition, the age‐specific issues it can cause and the availability of YOD services was identified as the main way to improve access to social care. This resonates with previous findings stressing the importance of YOD‐specific training and education for professionals [22]. Improving YOD awareness could also facilitate timely diagnosis [35] and consequently access to social care via more timely referrals.

The findings illustrate the need for improvements in service commissioning and care pathways. People with YOD and their families often struggle to find age‐appropriate services [16], and this is perhaps not surprising given our finding that many areas do not have agreed care pathways specifically for people with YOD and their families. Having dedicated YOD services was identified in our survey as an area of good practice and is consistent with previous findings that people with YOD are most satisfied with care provided by specialist YOD services [17]. YOD‐specific care pathways need to be built around the needs that commonly arise in association with YOD; for example, flexibility in offering services outside working hour [16]s and recognising that people may also have childcare responsibilities [20]. Peer support, identified as an area of good practice in the survey, should also be integrated into care pathways. Having the opportunity to meet with others in a similar situation is particularly valued by people with YOD and their families [20]. Support for people with YOD and their families should also be integrated within other services, as multi‐disciplinary working was identified as an area of good practice in our survey. This tallies with other evidence suggesting joint working involving co‐location or joint activities could improve post‐diagnostic support in dementia [36].

This study has certain limitations. The survey was restricted to people working in England because the organisation of social care provision differs in other areas of the UK. This limits the applicability of the findings to the rest of the UK and other countries. Despite multiple means used to promote the survey, response rates could have been higher given the size of the overall social care workforce which is approx. 1.705 million [37]. However, those who responded were from a good spread of roles and sectors. Respondents tended to be female and White British, reflecting the overall social care workforce in England whereby 79% are female and 68% are White [37]. There may have been some selection bias in that individuals with a particular interest in or significant involvement YOD may have been more likely to respond. The survey was designed to be short, but this limited the breadth of the questions and we were unable to ask participants follow‐up questions. Two of the questions had short four‐point Likert scales for participants to complete. These were kept short for simplicity and ease of completion, but a longer Likert scale may have allowed greater granularity in the data.

In conclusion, our survey findings highlight areas of good practice and areas for improvement in social care provision for people with YOD and their families. In particular, the findings highlight that the system is often reactive in responding to crises rather than being preventive, and that referrals may be triggered more often in response to carers' needs than by those of the person with YOD. The age‐specific challenges faced by those with YOD and their families require a tailored approach. Existing areas of good practice such as multi‐disciplinary and multi‐agency working, peer support, and dedicated YOD services could be built upon. However, the post‐diagnostic support system in the UK is often fragmented and not age‐appropriate, and provision varies across locations. Further improvements are required to ensure equitable access to all.

## Author Contributions

Conception and design—all. Analysis and interpretation of data—C.Q., H.Y., J.O. Drafting the manuscript—C.Q. Critical appraisal and review of the manuscript—all. Final approval of the manuscript to be published—all.

## Conflicts of Interest

The authors declare no conflicts of interest.

## Supporting information


Supporting Information S1

## Data Availability

The data that support the findings of this study are available from the corresponding author upon reasonable request.
