# Supplementary material for: Professionals' Views on Social Care Planning and Provision for People With Young‐Onset Dementia and Their Families in England: Findings From the DYNAMIC Study
Source: Int J Geriatr Psychiatry. 2025 Sep 20;40(9):e70155. doi: 10.1002/gps.70155 (PMC12449706; doi:10.1002/gps.70155)
Supplement: Supplementary file 1 — Supporting Information S1 [file GPS-40-e70155-s001.docx]

**DYNAMIC survey questions**

| 1. Please provide your current job title(s): |
| --- |
| 1. How long have you been in your current job role(s)? 2. What type(s) of service do you work within?   Local Authority Mental Health Trust  Primary Care Third Sector Organisation (charity)  Hospital Trust Integrated Care Board  Other (please specify) ___________________________________   1. How has/does your job role/personal experience give you contact/involvement with people with dementia? (Please do not use abbreviations in your response.) |
| 1. In which region of England do you predominantly work |
| 1. Roughly how many people with dementia have you had contact with over the previous 3 months? |
| 6a) Roughly how many of these people had young onset dementia (where the first symptoms occur under the age of 65)? |
| 1. Do you refer people with young-onset dementia or their families for:   (Please tick the box either yes/no)  social care assessments  **YES NO 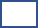**  carer assessments **YES 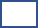 NO 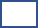**  social prescription **YES 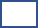 NO 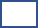**    Other (if yes, please specify) **YES 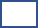 NO 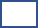** |
| 1. Do *you* undertake or have oversight of social care assessments, carer assessments or social prescription for people with young-onset dementia or their families? (Please circle your response)   **YES NO** |
| 1. Do you devise care plans or provide social care for people with young-onset dementia or their families, for example, related to finances, employment, children/family issues or other? (This could include having oversight of these aspects of social care.)   **YES NO** |
| 1. What types of situations might trigger a referral for social care assessment for people with young-onset dementia or their families?   __________________________________________________________________ |
| 1. Where you work, is there an agreed care pathway for people living with young onset dementia and their families?   **YES NO UNSURE**   1. Where you work, do you have a written policy that gives guidance on how to work with people with young onset dementia and their families? (Please circle your response)   **YES NO UNSURE** |
| 12a. If yes, please can you expand on your answer:  __________________________________________________________ |
|  |
| 12b. Do you feel these pathways are appropriate for people with young-onset dementia and their families?  __________________________________________________________________ |
|  |
| 13. How knowledgeable do you feel about social care planning and provision for people with young-onset dementia and their families?  **Very knowledgeable**  **Quite knowledgeable**  **Not very knowledgeable**  **Not at all knowledgeable**  **Don’t know** |
| 14. How confident are you in your ability to undertake social care planning and provision for people with young-onset dementia and their families?  **Very confident**  **Quite confident**  **Not very confident**  **Not at all confident**  **Not part of my role**  **Don’t know** |
|  |
| 15. Do you have any examples of good practice in social care planning and provision in dementia that you would like to share? Or examples from other areas that could be relevant? (Please keep these anonymous) |
|  |
| 16. Are you aware of any existing services/pathways that could be (or are already being) adapted for people with young-onset dementia? If so, please tell us about them.  __________________________________________________________________  __________________________________________________________________ |
|  |
| 17. How could access to social care for people with young-onset dementia be improved? |

18. Is there anything else you want to tell us?

**Some information about yourself:**

1. What is your gender?
2. What is your age?

20-30 31-40 41-50 51-60 61-70 71+

1. What is your ethnic group?
2. Do you consider that you work in an urban or rural location?
   **Urban Semi-Rural Rural All**
3. Have you ever undertaken any training/education about working with people with dementia? (Please circle your response)

**YES NO**

1. Do you access resources relating to young-onset dementia?

**YES NO**
